# Statin-Induced Hyper-Acute Reversible Liver Failure: A Case Report

**DOI:** 10.7759/cureus.103591

**Published:** 2026-02-14

**Authors:** Abdulaziz A AlTurki, Khaled AlQahtani, Abdulkreem A Alnasser, Meshal M AlTalha, Ali S AlHamdan

**Affiliations:** 1 Internal Medicine, King Saud Medical City (KSMC), Riyadh, SAU; 2 Gastroenterology and Hepatology, King Saud Medical City (KSMC), Riyadh, SAU; 3 Gastroenterology, King Saud Medical City (KSMC), Riyadh, SAU; 4 Adult Infectious Diseases, King Saud Medical City (KSMC), Riyadh, SAU

**Keywords:** acute/chemically induced, atorvastatin calcium/adverse effects, drug-induced liver injury/chemically induced, hydroxymethylglutaryl-coa reductase inhibitors/adverse effects, liver failure

## Abstract

Statins, particularly high-dose atorvastatin, are widely used for cardiovascular prevention but can rarely cause severe hepatotoxicity. We report a case of a male in his seventies who developed hyperacute hepatic failure within 24 hours of initiating atorvastatin 80 mg for secondary stroke prevention. Extensive investigations ruled out alternative causes, and his condition rapidly improved after discontinuation, implicating drug-induced liver injury. This case highlights the unpredictable nature of statin-induced hepatotoxicity, emphasizing the need for vigilance, particularly in elderly patients with comorbidities. Further research is needed to guide safe reintroduction strategies and alternative lipid-lowering options for affected individuals.

## Introduction

As defined in the literature [1], statins, or 3-hydroxy-3-methylglutaryl coenzyme A (HMG-CoA) reductase inhibitors, are among the most widely prescribed medications worldwide because of their proven efficacy in reducing cardiovascular morbidity and mortality. Atorvastatin effectively lowers total cholesterol and low-density lipoprotein (LDL) levels, thereby reducing the risk of atherosclerotic cardiovascular disease. Although statin-associated liver injury is uncommon, it remains a recognized adverse effect with mechanisms that are not yet fully understood. Atorvastatin undergoes extensive hepatic metabolism, primarily via the cytochrome P450 3A4 (CYP3A4) enzyme system, with subsequent elimination mainly through biliary and renal pathways. The reported incidence of statin-induced liver dysfunction varies, with most cases characterized by mild and transient elevations in liver enzymes. Clinically significant hepatotoxicity is rare and has been more frequently reported with higher doses, particularly 80 mg daily. The onset of liver injury is variable, most commonly occurring within the first six months of therapy or following a dose escalation, although delayed presentations after prolonged use have also been described [2-4,5,6].

## Case presentation

**Figure 1:**
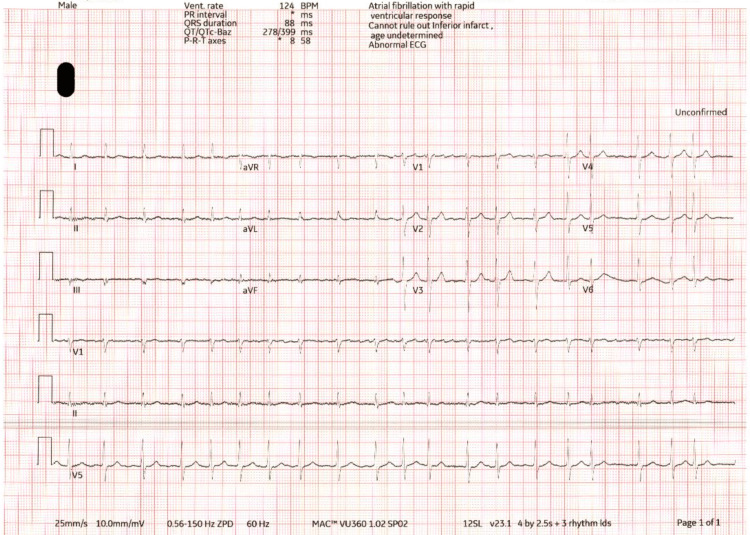
Twelve-lead ECG demonstrating atrial fibrillation with rapid ventricular response. ECG, electrocardiogram

We present a case of a male patient in his seventies of Middle Eastern descent, known to have poorly controlled type 2 diabetes mellitus on metformin and hypertension on losartan, with a history of a prior transient ischemic attack two years ago that was not treated. He did not have a history of herbal consumption, including khat (a common herb in Yemen containing amphetamine), and, for religious beliefs, he never consumed alcohol. Our patient was brought to our Emergency Department after his family noted left-sided weakness and slurred speech, first noticed when he woke up on the morning of presentation. On examination in the Emergency Department, his vital signs were HR 92 bpm, BP 155/103 mmHg, O₂ saturation 95% on room air, RR 24, and temperature 36.5 °C. His body mass index (BMI) was 35.55 kg/m². Neurologically, his Glasgow Coma Scale (GCS) was 14/15; he was fully conscious, with dense left hemiplegia, dysarthria, and facial drooping. His peripheral pulse was irregular, and a 12-lead ECG confirmed newly found atrial fibrillation (Figure 1).

Physical examination showed central obesity. There was no scleral icterus, no visible jaundice, and no palpable organomegaly. His laboratory assessment upon admission is shown in Table 1.

**Table 1:** Laboratory results upon admission. ALT, alanine aminotransferase; AST, aspartate aminotransferase; ALP, alkaline phosphatase; BUN, blood urea nitrogen; RDW-CV, red cell distribution width (coefficient of variation); MPV, mean platelet volume; MCHC, mean corpuscular hemoglobin concentration; MCV, mean corpuscular volume; MCH, mean corpuscular hemoglobin

Test (Unit)	Result	Reference range
WBC (×10³/µL)	17.1	4-11
Hemoglobin (g/dL)	13.5	13.5-18.0
Hematocrit (%)	40.3	42-54
MCV (fL)	88.9	76-96
MCH (pg)	29.7	27-32
MCHC (g/dL)	33.4	31.0-35.5
MPV (fL)	11.0	7.4-10.4
Platelets (×10³/µL)	247	150-400
Neutrophils # (×10³/µL)	15.2	2.0-7.5
Lymphocytes # (×10³/µL)	1.27	1.0-4.4
Monocytes # (×10³/µL)	0.45	0.1-1.1
Eosinophils # (×10³/µL)	0.00	0.1-0.5
Basophils # (×10³/µL)	0.01	0-0.1
RDW-CV (%)	15.3	11.5-14.5
Creatinine (µmol/L)	132.4	63.6-110.5
BUN (mmol/L)	11.8	3.0-9.2
Sodium (mmol/L)	137	136-145
Potassium (mmol/L)	5.3	3.5-5.1
Chloride (mmol/L)	101	98-107
ALT (U/L)	42	0-55
AST (U/L)	34	11-34
Total bilirubin (µmol/L)	20.8	5.1-20.5
Direct bilirubin (µmol/L)	10.3	
ALP (U/L)	81	50-116

**Figure 2:**
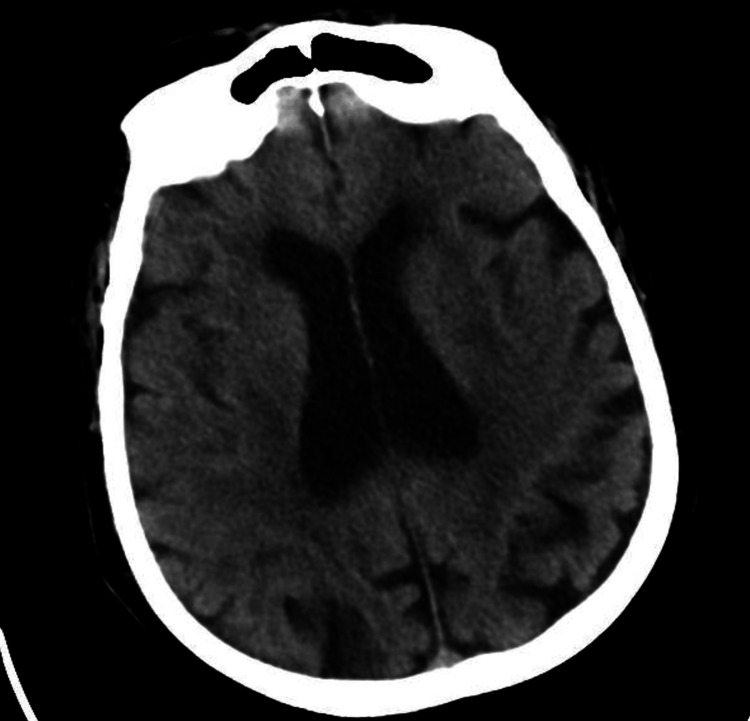
Plain CT scan of the head demonstrating senile atrophy.

A CT scan of his brain revealed Senile diffuse atrophic/chronic microangiopathic changes (Figure 2).

**Figure 3:**
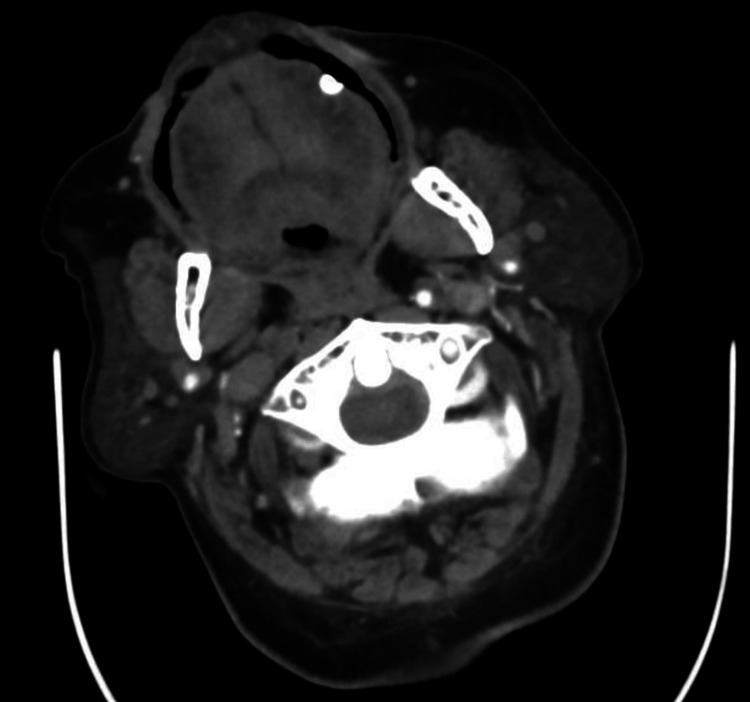
CT angiogram (axial view) showing the right ICA with non-opacification from its proximal portion to the clinoid segment intracranially. ICA, internal carotid artery

**Figure 4:**
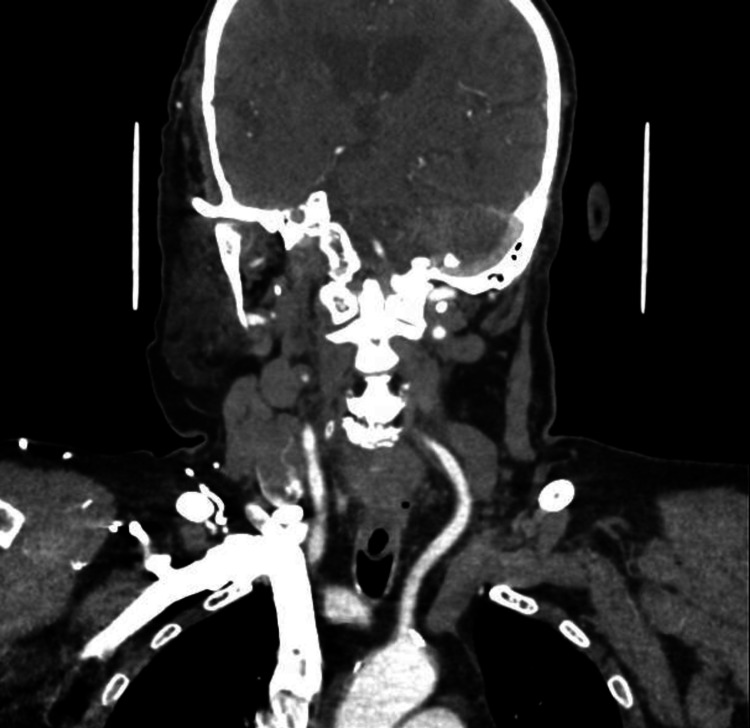
CT angiogram (sagittal view) showing the right vertebral artery with significant stenosis at its origin.

**Figure 5:**
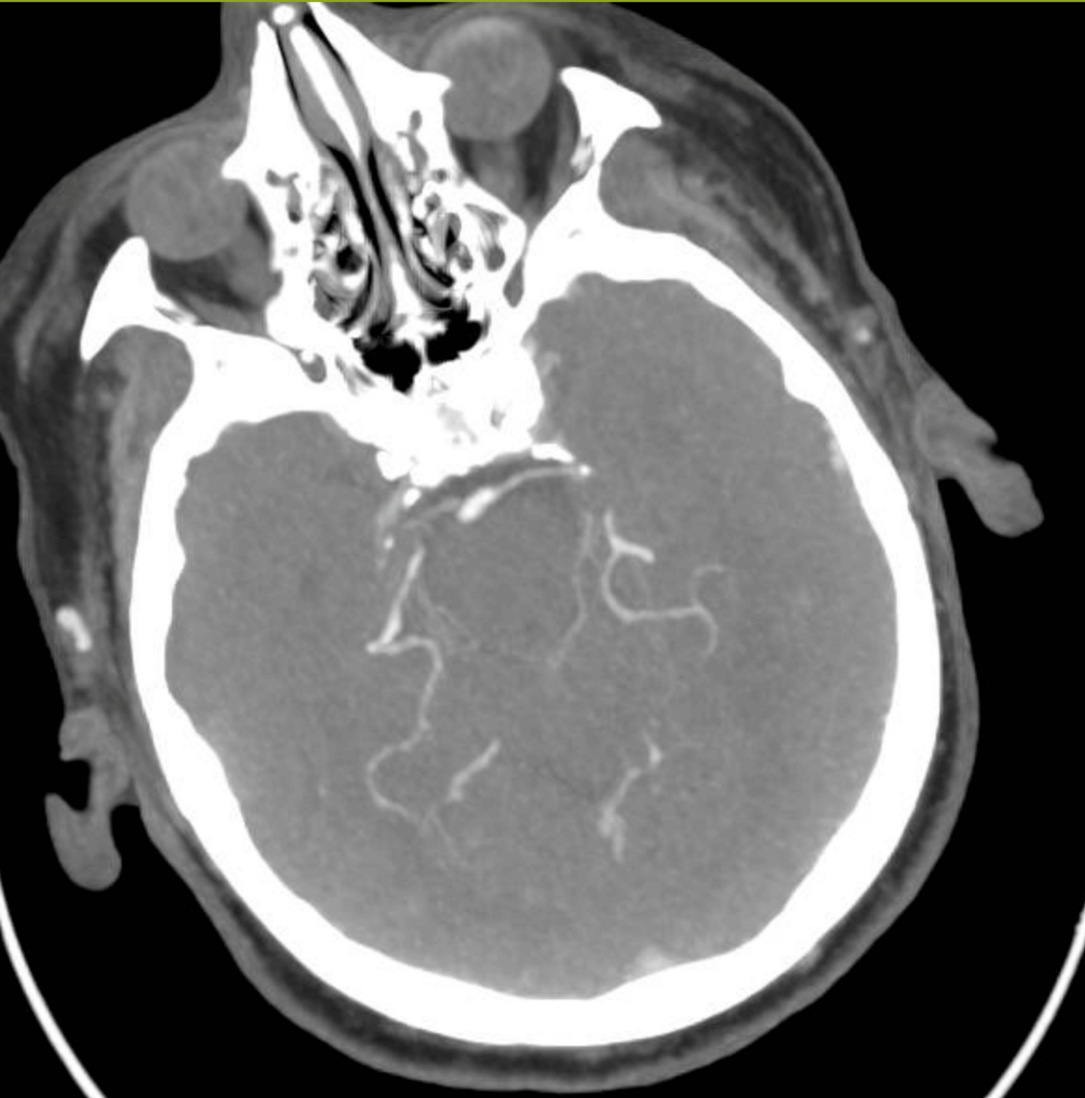
CT angiogram (axial view) showing narrowing of the P2 segment of the left PCA. PCA, posterior cerebral artery

Our neurology service requested a CT perfusion study to evaluate his case for mechanical thrombectomy; the study revealed atherosclerotic changes most severely involving the right internal carotid artery (ICA), which showed non-opacification from its proximal portion to the clinoid segment intracranially, as well as a right vertebral artery with significant stenosis at its origin and in the P2 segment of the left posterior cerebral artery (PCA). There were also small, faint hypodensities at the right external capsule and left corona radiata, likely ischemic changes of indeterminate age and chronicity (Figures 3-5).

As such, he was deemed unfit for mechanical thrombectomy, nor was he a candidate for receiving tPA. Our patient was admitted to our High Dependency Stroke Unit as a case of ischemic stroke outside the therapeutic window for treatment, for further workup.

**Figure 6:**
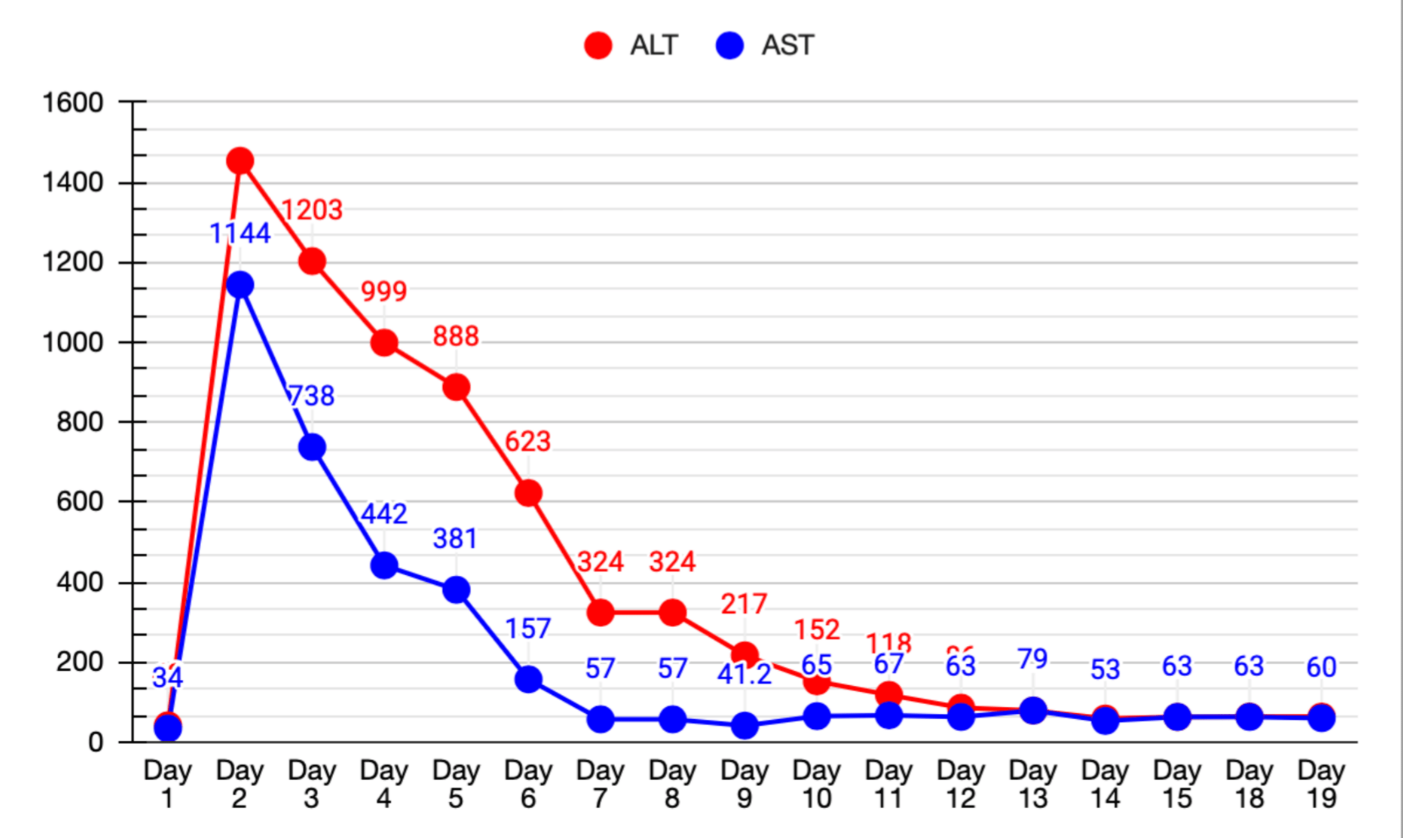
Alanine aminotransferase (ALT) and aspartate aminotransferase (AST) trend. Line graph showing serum ALT and AST levels (U/L) plotted against time (days).

**Figure 7:**
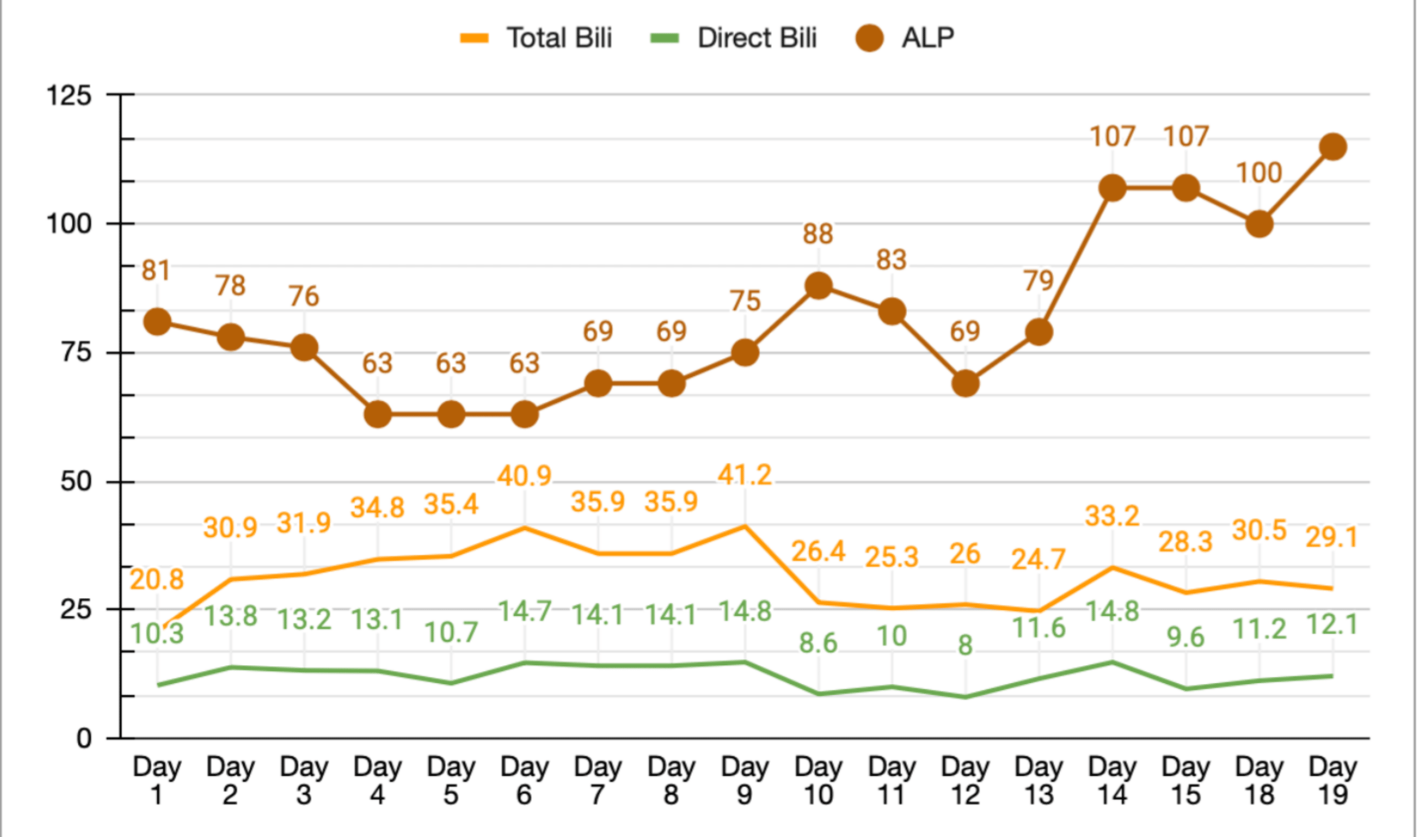
Total bilirubin, direct bilirubin, and alkaline phosphatase trend. Line graph showing serum total bilirubin, direct bilirubin, and alkaline phosphatase levels plotted against time in days.

He was subsequently commenced on atorvastatin 80 mg and aspirin 100 mg for secondary prevention, in addition to oral omeprazole 20 mg once daily for gastrointestinal prophylaxis and stress ulcer prevention. Bisoprolol was started for tachycardia, and later during his inpatient stay, apixaban was introduced for atrial fibrillation. Metformin was withheld during his hospitalization, and an insulin aspart sliding scale was initiated. Permissive hypertension was allowed for the first 24 hours. Twenty-four hours after admission (day 2), his mental status suddenly deteriorated; he became obtunded and difficult to arouse, with a GCS of 9/15. He was jaundiced and had right upper quadrant tenderness. His LFTs rose markedly from normal to an ALT of 1454 U/L and an AST of 1148 U/L, with a total bilirubin of 30.9 µmol/L and a direct bilirubin of 13.8 µmol/L (Figures 6-7).

**Figure 8:**
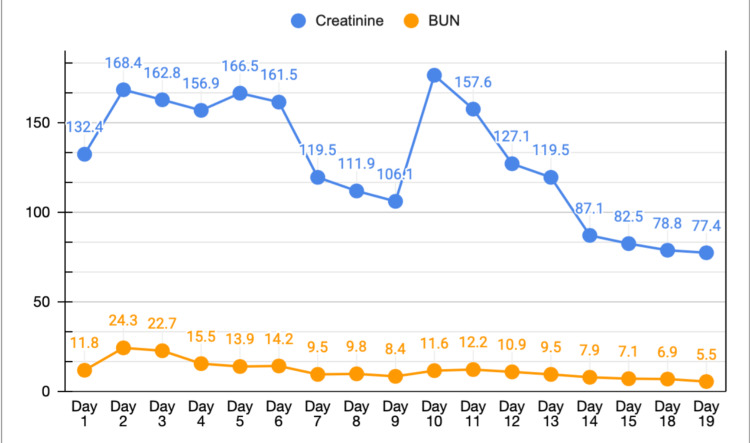
Creatinine and blood urea nitrogen (BUN) trend. Line graph showing serum creatinine and BUN levels plotted against time in days.

With the sudden derangement in his liver function, there was also a concurrent acute kidney injury; his creatinine and blood urea nitrogen increased to 168.4 µmol/L and 24.3 mmol/L, respectively, on day 2 (Figure 8).

**Figure 9:**
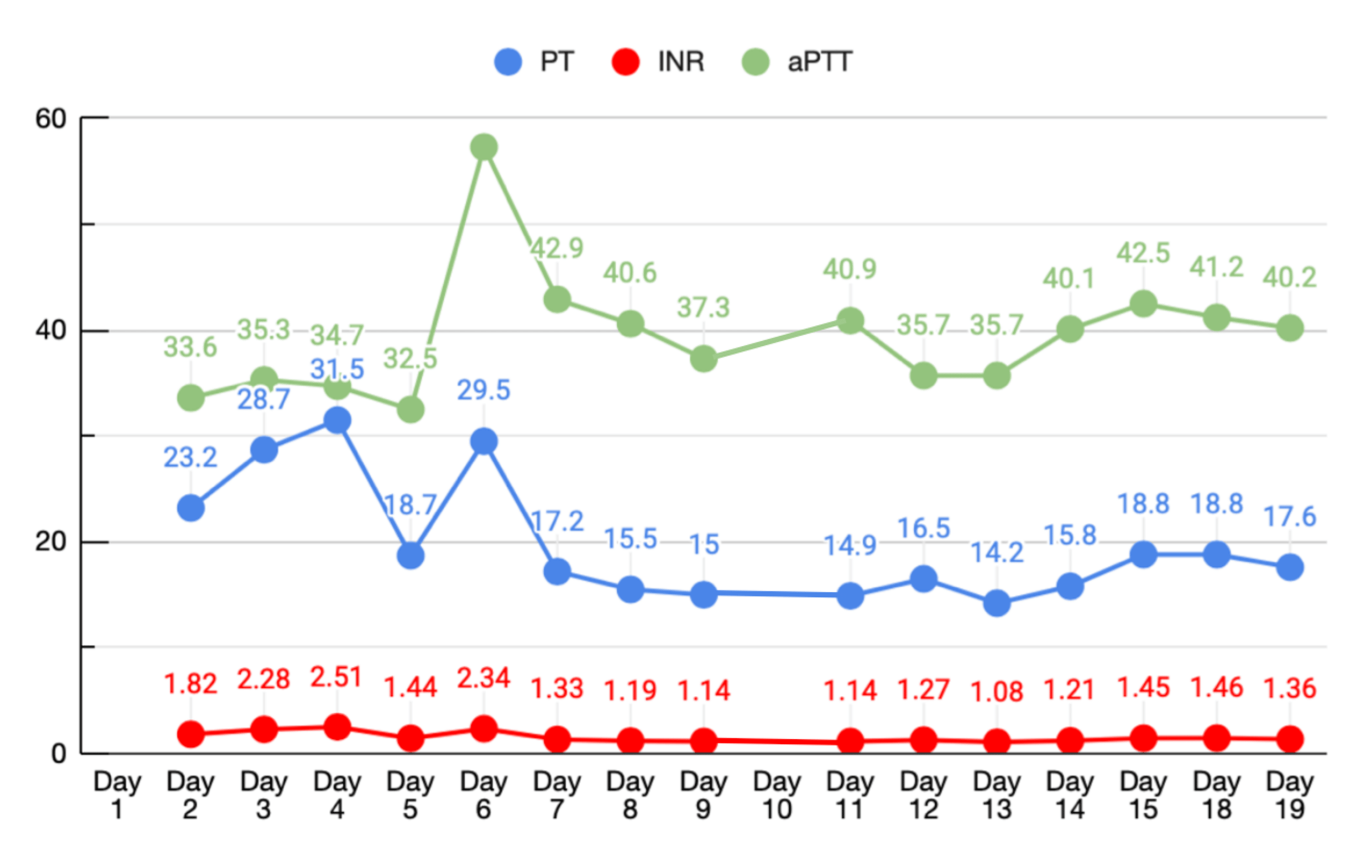
Prothrombin time (PT), partial thromboplastin time (PTT), and international normalized ratio (INR) trend. Line graph showing serial measurements of PT, PTT, and INR plotted against time.

Lastly, our patient’s coagulation profile increased on day 3, with a prothrombin time (PT) of 28.7 seconds and an international normalized ratio (INR) of 2.28, but a normal activated partial thromboplastin time (aPTT) of 35.3 seconds (Figure 9). Intermittently throughout his stay, multiple venous blood gases were obtained to monitor for any sequelae of renal injury or liver failure causing metabolic acidosis. However, the patient maintained near-normal values of pH, carbon dioxide, lactate, and sodium bicarbonate.

**Figure 10:**
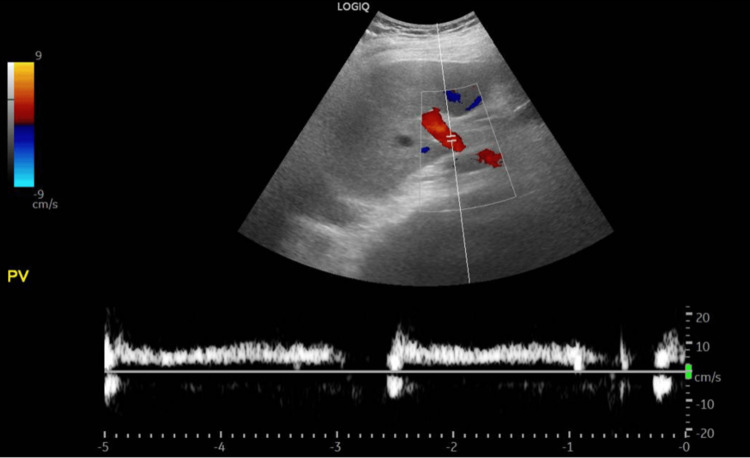
Liver ultrasound with Doppler. Grayscale and Doppler ultrasound images of the liver demonstrating normal hepatic parenchyma and normal hepatic vascular flow.

**Figure 11:**
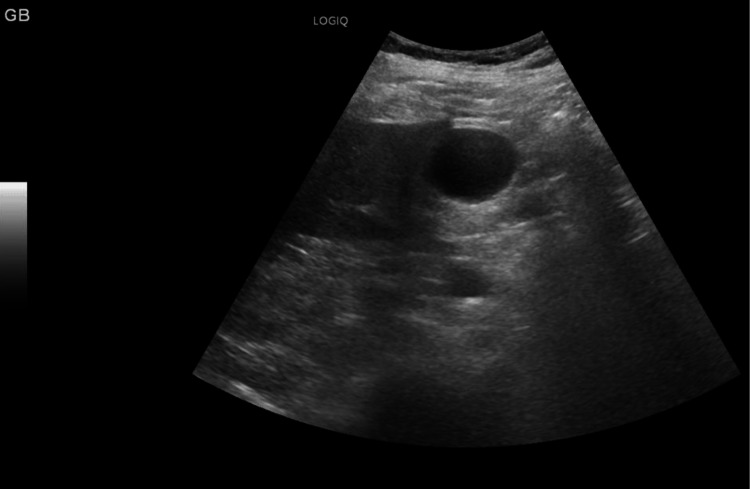
Gallbladder ultrasound. Grayscale ultrasound image demonstrating a normal gallbladder without wall thickening, gallstones, or pericholecystic fluid.

There were no recorded incidences of hypotension during his stay, no spikes of fever, no nausea/vomiting, no diarrhea, and no change in stool or urine color. After this event, we reviewed all of his medications and made adjustments. Due to his liver injury, atorvastatin was withheld until a more detailed assessment was completed. Septic screening with sputum, blood, and urine cultures, along with urinalysis, was sent, and he was empirically treated with piperacillin/tazobactam until culture results were available. We considered that the acute elevation might have been due to a thromboembolic event such as hepatic vein thrombosis; a liver ultrasound with Doppler was arranged, but the study came back normal with patent portal and hepatic veins (Figure 10). A gallbladder scan showed no stones (Figure 11).

**Figure 12:**
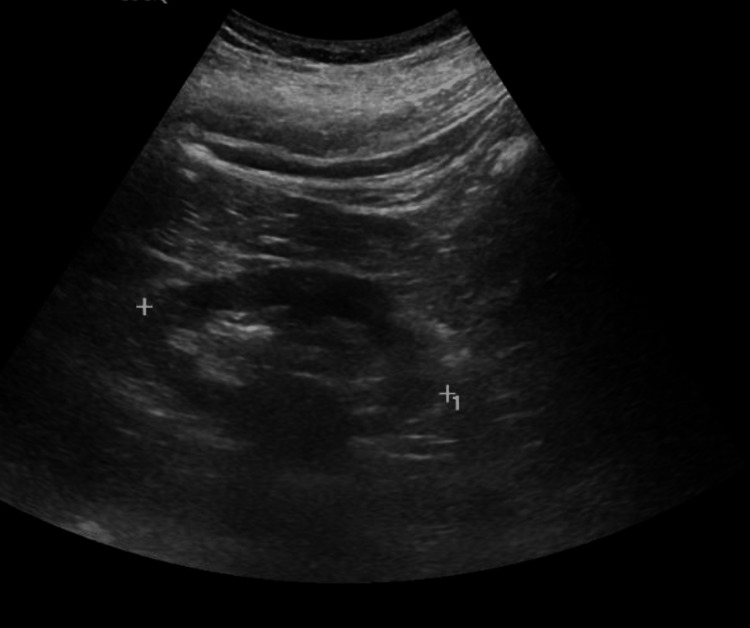
Ultrasound of the left kidney. Grayscale ultrasound image of the left kidney demonstrating grade II increased echogenicity.

**Figure 13:**
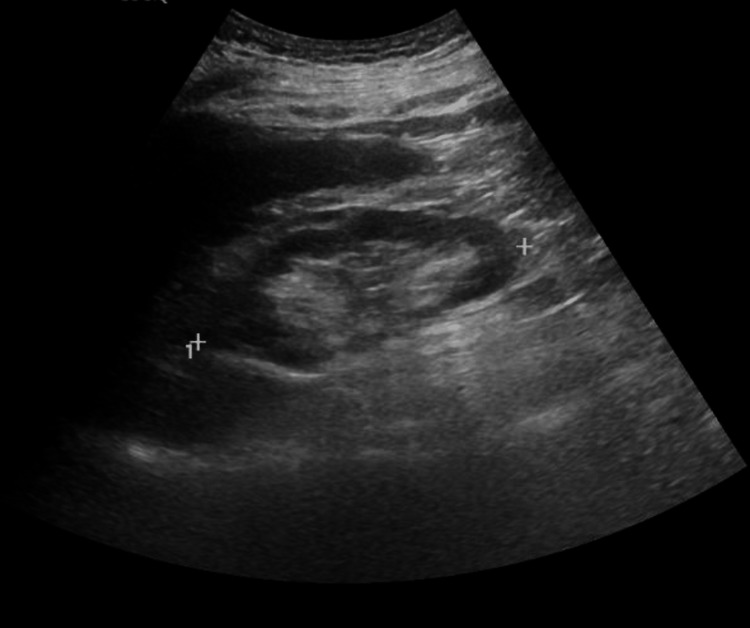
Ultrasound of the right kidney. Grayscale ultrasound image of the right kidney demonstrating grade II increased echogenicity.

Another possibility was viral hepatitis, but testing for hepatitis B and C as well as HIV was negative. An autoimmune panel, including anti-mitochondrial antibodies, ceruloplasmin level, anti-smooth muscle antibodies, and ANA, was also negative. Moreover, IgA, IgG, and IgM titers were within the normal range. In addition, thyroid function testing, iron studies, and creatine kinase were normal. Acetaminophen and blood alcohol levels were undetectable. His sputum, blood, and urine cultures with urinalysis were unremarkable, and antibiotics were subsequently discontinued. A kidney ultrasound was also performed to evaluate the cause of his acute kidney injury; it revealed grade II increased echogenicity, suggestive of chronic renal impairment, with no other abnormalities found (Figures 12-13). Ultrasound of the left kidney showing grade II echogenicity.

What was remarkable was the timing of the discontinuation of atorvastatin, which was the most beneficial adjustment we made for our patient. The day after withholding atorvastatin, his clinical status gradually yet substantially improved. AST and ALT trended down to a normal baseline. His liver function tests then shifted from a hepatocellular pattern of injury to a cholestatic pattern, a common pattern shift seen in drug-induced liver injury. His kidney function tests and coagulation profile also recovered.

We concluded that his presentation most likely represented severe drug-induced liver failure, as all other possibilities were excluded. After he achieved recovery, he was eventually discharged home with no further sequelae of liver failure or injury and with normal liver function tests. Since the patient would have benefited from statin therapy, our plan was for him to follow up in our hospital’s outpatient department after discharge to restart a lower dose of atorvastatin or initiate an alternative such as rosuvastatin. Unfortunately, he was lost to follow-up, and we were unable to continue treatment.

## Discussion

The authors report a case of a patient who developed acute hepatic failure after initiating high-dose statin therapy (atorvastatin 80 mg orally once daily) as secondary prophylaxis following an acute ischemic stroke. All other differential diagnoses were ruled out with thorough investigations, and the patient was diagnosed with drug-induced liver failure. His condition began to improve immediately after discontinuation of the high-dose statin.

It has been known that statins rarely and unpredictably cause hepatic failure. As affirmed by the Statin Liver Safety Task Force, liver function monitoring is not recommended while on statin therapy, and drug-induced liver injury is diagnosed by exclusion [2]. Although rare, atorvastatin has been shown to cause acute hepatic failure in a small number of cases, such as ours. Atorvastatin and simvastatin were described to cause drug-induced liver injury with cholestatic and hepatocellular patterns, respectively, in approximately 1-3% of cases, though not to the extent of hepatic failure [3]. There have been multiple extensive studies regarding the association of statins with liver injury but not hepatic failure [4-6]. Only a few case reports have described severe drug-induced liver injury [3,7-9], in addition to our case. A systematic review estimated that hepatic failure occurs at a rate of 1 per million person-years of use [10]. Therefore, it is important to be aware of this rare complication, as statins are common in various clinical situations. Liver toxicity is reversible after removal of the offending agent; therefore, depending on the circumstances, discontinuation of the offending agent (statin) may precede thorough investigations, as the diagnosis of drug-induced liver injury may be delayed. Statins are generally well tolerated, although statin-related adverse events are dose dependent, including liver injury, as described in different studies [11-13]. The latency from statin initiation to the onset of liver injury varies widely, ranging from 1-12 months to more than a year [14], unlike in our case, where the temporality of statin-induced liver failure was acute and occurred within days. This indicates the unpredictability of liver damage, which cannot be controlled. In light of this information, the use of high-dose statins may correlate with a higher likelihood of potential liver injury, which might progress to liver failure.

According to the American Heart Association/American Stroke Association guideline for the prevention of stroke in patients with stroke and transient ischemic attack, initiation of high-dose statins such as atorvastatin 80 mg is strongly recommended to reduce stroke recurrence [15]. This recommendation is based on two randomized clinical trials, Stroke Prevention by Aggressive Reduction in Cholesterol Levels (SPARCL) and Treat Stroke to Target (TST), in addition to multiple smaller studies [16,17].

An ESC/EAS guideline for the management of dyslipidemia recommends that statin therapy in elderly individuals aged >75 years should be initiated at the lowest tolerated dose and then titrated upward to avoid myopathy. This recommendation is largely based on the usual health status of geriatric patients, who often take multiple medications and therefore have an increased risk of drug-drug interactions, as well as possible pre-existing renal impairment, which increases the risk of statin-related adverse effects. However, there is no clear guideline recommendation for reducing statin doses in elderly populations to mitigate liver injury [18].

Additionally, to the best of our knowledge, no guideline or institutional protocol defines the methodology for re-challenging patients who have developed statin-related liver failure with statin therapy again. From our experience with this case, this approach should be undertaken with great caution, as it may carry significant morbidity and mortality.

## Conclusions

This case highlights the rare but serious risk of acute hepatic failure associated with high-dose atorvastatin therapy. While statins are generally well tolerated and widely recommended for secondary prevention of cardiovascular events, their potential for severe liver injury, though uncommon, should not be overlooked. Our patient’s rapid clinical improvement following statin discontinuation strongly supports drug-induced liver injury as the underlying cause. Given the unpredictable nature of statin-related hepatotoxicity, clinicians should maintain a high index of suspicion when patients on statin therapy present with acute liver dysfunction.

Currently, there are no established guidelines for reintroducing statins in patients who have experienced statin-induced liver failure. This case underscores the need for individualized risk assessment and cautious re-challenging, particularly in elderly patients with multiple comorbidities. Further research is warranted to develop standardized protocols for managing statin-related hepatotoxicity and determining safe alternatives for lipid-lowering therapy in affected individuals.
